# Profiling single-cell level phagocytic activity distribution with blood lactate levels†

**DOI:** 10.1039/d1ra02759j

**Published:** 2021-06-16

**Authors:** Kurt Wagner, Muhammad A. Sami, Corey Norton, Jonathan McCoy, Umer Hassan

**Affiliations:** Department of Biomedical Engineering, School of Engineering, Rutgers, The State University of New Jersey Piscataway NJ 08854 USA; Department of Electrical and Computer Engineering, School of Engineering, Rutgers, The State University of New Jersey Piscataway NJ 08854 USA umer.hassan@rutgers.edu +1-848-445-2164; Department of Emergency Medicine, Robert Wood Johnson Medical Hospital, Rutgers, The State University of New Jersey New Brunswick NJ 08901 USA; Global Health Institute, Rutgers, The State University of New Jersey New Brunswick NJ 08901 USA

## Abstract

The ability to kill infecting microbes is an essential facet of our immune response to an infection. However, phagocytic ability is often overlooked as a part of immunological profile in infected patients' diagnosis, as the understanding of phagocytic capabilities in disease states is incomplete. In this work, we have evaluated for the first time the relationship between blood lactate level and the neutrophil phagocytic activity at a single-cell level. Blood samples (*N* = 19) were grouped on the basis of their blood lactate levels *i.e.*, below (control) or above 2 mmol L^−1^ (high-risk) (*i.e.*, 2 mmol L^−1^ is a common clinical lactate threshold used for patients' triage). Neutrophils were isolated from whole blood and then incubated with fluorescent IgG coated beads for 40 minutes, and the ability of each neutrophil to internalize beads was quantified. Single-cell phagocytic activity analysis has shown interesting findings such as: (i) a single neutrophil was able to internalize up to 7 beads, (ii) for a control group, 39.76% cells didn't internalize any beads, while for a high-risk group, 30.65% cells didn't show any phagocytic activity, (iii) similarly, 30.46% cells internalize only 1 bead in a control group, while for a high-risk group the activity is slightly higher with only 31.73% cells showing single bead internalization, and (iv) 7 bead internalization activity was much higher for samples in a high-risk group (0.6% cells) compared to a control group (0.17% cells). We used multiple statistical tests to compare these differences. For a two-tailed *T*-test, we used the mean phagocytic activity of the cells (*i.e.*, the average number of beads internalized by cells) isolated from the blood samples in the two groups (1.14 *vs.* 1.35) and found the *p*-value to be 0.08. We also used principal component analysis (PCA) on this high dimensional phagocytic activity distribution data and performed dimension reduction. However, the first 3 principal components didn't show a clear distinction between groups. Next, we developed machine learning models using artificial neural networks (ANNs) to differentiate between the distribution of phagocytic activity in neutrophil populations of the two groups. Our models yielded area under curve (AUC) values below 0.7 for receiver operator characteristic curves. Although our study highlighted interesting phagocytic activity findings at a single cell level, it further highlights the need for integration of an individual patient's medical record to get more personalized insights into individual phagocytic activity in the future.

## Abstract 2

Investigating the relationship between neutrophil phagocytic activity and blood lactate levels by employing single-cell data.

## Graphical abstract

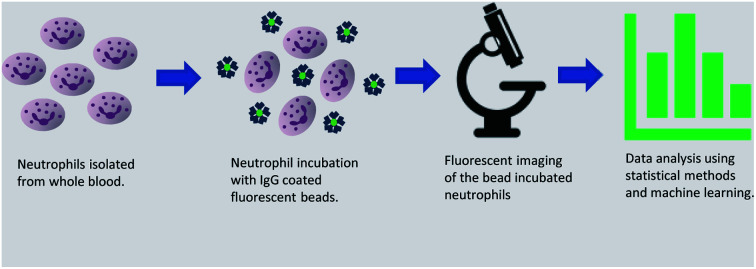

## Introduction

Phagocytosis has long been a topic of interest due to the important role it plays in innate immunity. Internalization of pathogens quells initial infections and leads to further stimulation of immune functions. This is especially relevant in sepsis, a life-threatening immune response to infection.^1,2^ Sepsis is a leading cause of mortality in the United States. In the United States alone, more than 1 million people develop this condition and about half a million perish.^3,4^

Sepsis can be caused by a variety of factors but the presence of foreign bacterial pathogens in the body is the most common one. Phagocytosis is one of the ways in which our body deals with these foreign pathogens, so a better understanding of this process can potentially help in making more informed decisions regarding septic patients. There are many aspects of phagocytic activity which have not been fully explored. To date, only limited research has been done regarding the potential applications of understanding phagocytic ability in disease states. There is a considerable amount of seemingly conflicting evidence about the changes in phagocytic activity throughout the progression of severe infections.^5,6^ Needless to say, the relationship between phagocytosis and sepsis needs to be understood with more clarity.

Many studies demonstrate a decrease in overall neutrophil functionality at the onset of sepsis. One possible explanation is the massive influx of immature neutrophils into the blood stream, a key feature of sepsis and systemic inflammatory response syndrome (SIRS) in humans as well. This phenomenon is widely documented and is included in most definitions of sepsis.^7–9^ A study of immature *versus* mature neutrophils, maturity being determined by levels of FcR CD16 expression, was conducted to evaluate the role that this recruitment of cells has on overall immune function. Neutrophils with lower levels of CD16 expression in sepsis and SIRS patients had lower phagocytic indices than mature neutrophils, measured by flow cytometry.^10^ Additional bacterial killing studies on agar plates supported the results from the flow cytometry, as immature neutrophils ingested and killed fewer bacteria compared to mature neutrophils.

Deficiencies in neutrophil function have been tied to other receptors as well. In cases of inflammation, the expression of CD64, a high affinity receptor to immunoglobulin G, is rapidly increased in the neutrophil population.^11^ It has been indicated that these CD64+ neutrophils may have reduced phagocytic activity in the first 48 hours of sepsis compared to healthy controls.^12^ After incubation with fluorescently labeled latex beads, the mean fluorescence intensity of neutrophils and monocytes was calculated. CD64+ neutrophils and monocytes both showed significantly lower fluorescence compared to controls. This decrease was even greater in patients who were also experiencing acute respiratory distress. The authors suggested that heightened levels of CD64 in inflammation may lead to overstimulation of immune cells, leading to lessened effectiveness. It was also noted that no significant difference in phagocytic index was found between survivors and non-survivors.

Contrary to these results, some studies have indicated increased rather than decreased phagocytosis at the onset of sepsis. One such study examined patients with severe sepsis and found that the average phagocytic activity was higher in septic patients compared to healthy controls. The work focused on the impaired chemotaxis of neutrophils, but a section which was intended to demonstrate functional actin polymerization showed that untreated neutrophils of septic patients had significantly higher phagocytic indices.^13^ There is only a limited explanation as to how this index was calculated, and it may mask potentially important aspects of the phagocytic ability of different cells. Another study found that the immune cells of patients collected within 48 hours of sepsis diagnosis had much higher average fluorescence compared to healthy controls when challenged with fluorescently labeled targets.^14^ However, there was a large variance for septic patients, covering a range twice as large as the control.

The wide variance of phagocytic ability in patients with severe infections was seen in numerous studies. In one such study, infected patients again showed higher phagocytic indices without reaching the level of statistical significance due to the spread of the data.^15^ Micromanipulation studies allowed for the direct comparison of healthy mature neutrophils and neutrophils of varying maturity from septic patients. Mature neutrophils from septic patients had the capability to internalize the greatest number of targets, followed by mature neutrophils of healthy patients, which were trailed by immature neutrophils from septic patients. This may offer an explanation as to how overall phagocytic function may vary in septic individuals despite consistently high neutrophil counts. Only in septic patients was there a noticeable portion (38%) of the neutrophil population which internalized less than three targets during the incubation period. In the healthy population, less than 3% fell into this category.

When studied as a single value for an individual's entire leukocyte population, phagocytic activity is tied to a plethora of conflicting studies. Two different patients can exhibit the same mean phagocytic activity even if one has a small number of neutrophils with very high activity and the other has a larger number of neutrophils exhibiting moderate level of activity. Therefore, it may be useful to also study the distribution of phagocytic activity across a patient's neutrophil population, in addition to combining it into a single value which can possibly result in the loss of meaningful data. In this study, we evaluated and compared the distribution of phagocytic activity in the neutrophil populations at a single-cell level from blood samples collected from 19 patients at Robert Wood Johnson Medical Hospital. The patients were grouped based on the basis of their blood lactate levels, a test which is commonly used to stratify risk for adverse outcomes for septic patients.^16^ Those with blood lactate of 2 mmol L^−1^ or higher were considered higher risk patients, and those with blood lactate less than 2 mmol L^−1^ were considered the normal or control group.^16,17^ Immune cells from these patients were challenged with fluorescently labeled targets and observed under a fluorescent microscope to quantify the distribution of phagocytic activity.^18,19^ This data was then analyzed using statistical tools, principal component analysis and MATLAB based machine learning methods to determine if a relationship existed between the distribution of neutrophil phagocytic activity and blood lactate levels for hospitalized patients.

## Materials and methods

### Human subject statement

This human subjects' study is approved by Institutional Review Board (IRB) at Rutgers, The State University of New Jersey and Robert Wood Johnson Medical Hospital (IRB application # Pro2018002356). The experiments were done according with the IRB guidelines. The blood samples were de-identified by the hospital staff before providing them to investigators. Blood sample acquisition for our experiments in this clinical study was done in accordance with the IRB protocol guidelines. Patients were selected for whom a lactate test was ordered, and we were provided de-identified left-over blood samples which didn't require the informed consent in accordance with the IRB guidelines.

### Patient population

A total of 19 de-identified human peripheral blood samples were received from Robert Wood Johnson Hospital, New Brunswick, NJ with each sample containing approximately 1 mL of whole blood. The time of admission, time of blood draw, and blood lactate levels were known for each sample. The patient population included individuals for whom a lactate blood test was ordered. Typically, patients who are recommended for these tests present indications of infection and sepsis, including fever and shortness of breath. Of the 19 blood samples, 8 had a lactate level below 2 mmol L^−1^ and the rest were above 2 mmol L^−1^.

### Neutrophil isolation from whole blood

Whole blood was diluted in a 1 : 1 with 1× phosphate buffered saline (PBS). The blood–PBS mixture was layered over Ficoll Paque Plus density gradient (product number: 17144002) in microcentrifuge tubes at the recommended ratio of 4 parts blood–PBS to 3 parts density gradients. The tubes were spun at 400 × *g* for 30 minutes at room temperature. During centrifugation, erythrocytes and polymorphonuclear leukocytes form a dense, red layer at the bottom of the tube. After centrifugation, all layers except erythrocyte pellet were aspirated and discarded. Red blood cells were then lysed *via* hypotonic shock by adding distilled H_2_O, and tonicity was restored within 20 seconds with the addition of 10× PBS. The tubes were centrifuged again at 400 × *g* for 10 minutes to pellet the remaining leukocytes. Supernatant containing cellular debris was discarded, before washing the pellet in 1× PBS, resuspending the cells, and repeating the centrifugation. Erythrocyte lysis was repeated if the pellet or liquid appeared dark red, to achieve higher purity. The remaining polymorphonuclear leukocytes (PMNL) were resuspended in RPMI 1640 Medium from Thermo Fisher Scientific (catalog number: 11875085) and counted using a hemocytometer. This protocol has been demonstrated to have a very high capture efficiency and purity for neutrophils.^20^ The process of isolating neutrophils from whole blood was effective. The purity is estimated to be above 95% based on literature^20^ and our manual observations of very few remaining erythrocytes or macrophages.

### Phagocytosis assay

**Fig. 1:**
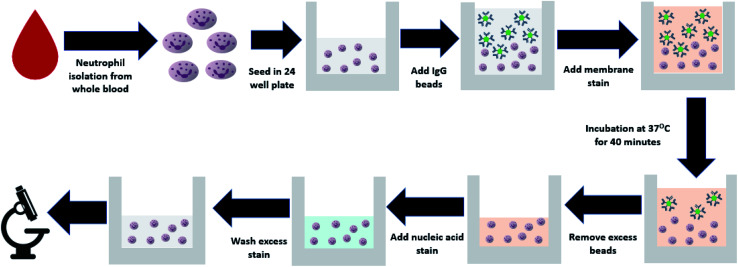
Flowchart explaining the protocol for neutrophil isolation from whole blood and the addition of fluorescent beads for quantification of phagocytic activity.

Neutrophils were seeded in a 24 well plate by adding ∼6 × 10^5^ cells per well, to reach a target density of 3 × 10^5^ cells per cm^2^. Each blood sample was run in triplicate, with 3 wells per patient sample. Fluorescently labelled polystyrene beads conjugated with human IgG antibody from Spherotech (product number: HFP-0852-5) were added to the wells in a cell to target ratio of 1 : 25. The beads are spheres, approximately 1 μm in diameter, similar to the size of common bacteria such as *E. coli* and *S. aureas*. CellBrite cytoplasmic membrane dye (catalog number: 30022) was added with the beads for later visualization of the cell surface. Cells were incubated at 37 °C to allow phagocytosis to occur. After 40 minutes, bead containing media was removed from the wells, and chilled 1× PBS was used to wash remaining beads from the wells before the addition of Hoescht nucleic acid stain from Thermo Fisher scientific (catalog number: H3570). Samples were kept on ice for 10 minutes before being washed with PBS to remove excess stain and imaged with a fluorescence microscope. The entire phagocytic assay from neutrophil isolation to the addition of fluorescent polystyrene beads is shown in Fig. 1. Furthermore, the approximate time required for each step involved in the protocol can be seen in Table S1.†

### Microscopic imaging of neutrophils and beads

Neutrophils incubated with the antibody tagged fluorescent beads were imaged using Olympus IX81 fluorescent microscope. Five locations in each of the triplicate wells per patient sample were imaged using both bright field and fluorescent microscopy. For the Hoescht 33342 nuclear stain (360–461 nm) blue settings were used, green for the labelled polystyrene beads (460–495 nm), and red for the CellBrite orange membrane dye (540–575 nm). Each location was set using a motorized stage and automated imaging procedure. As such, some images were out of focus and were excluded from analysis.

### Image processing

ImageJ was used for image processing. The images collected from the fluorescent microscope were first converted into binary images, as seen in Fig. S1.† The cytoplasmic membrane dye was used to establish the area of cells in the image and define the regions of interest for further analysis. Blue nucleic acid stain was then used to confirm the locations and count of cells that were first obtained from the orange membrane stain. Furthermore, the blue nucleic acid stain also allowed for exclusion of dead cells and artifacts present in the fluorescent membrane images. The nuclei of dead cells fluoresce brighter than those of live cells due to the compromised cellular membranes and nuclear envelopes as seen in Fig. S2.† Cells which exhibited excessive blue nuclear fluorescence were considered dead and were excluded from further analysis. Regions which fluoresced orange with the membrane stain but without blue fluorescence of DNA were considered noise, thought to be the result of membrane stain adhering to the surface after cells were sheared off during washing steps. The beads inside the final regions of interest determined by both the membrane stain and the nuclear stain were counted as internalized particles, and the number of internalized beads was counted for each cell. All counts were performed manually, and this process was repeated for each image, resulting in 5 images per well, and a total of 15 images per sample. The flowchart shown in Fig. S3† represents the steps involved in the image processing protocol.

### Phagocytic activity distribution

For each patient, the phagocytic activity distribution for the neutrophil population was created by stratifying the imaged neutrophils based on the number of beads they had internalized. This resulted in the creation of a univariate frequency distribution for each patient, showing the number of neutrophils which had internalized 0 through 7 beads. Finally, we divided each frequency with the total number of observed neutrophils to get the proportion of a patients' neutrophil population which had internalized any specific number of beads ranging from 0 to 7.

### Statistical analysis

We calculated the mean neutrophil phagocytic activity for each patient by getting the average number of beads internalized per neutrophil. Statistical analyses were then used to compare the mean phagocytic activity of the patients in the two groups. First, Shapiro–Wilks test was applied on each data set to determine its normality. Bartlett's test was then used to determine if there was any significant difference between the variances of the data sets. A two-tailed *T*-test was finally used to compare the means of the two data sets. A significance level of (*α* = 0.05) was used. These statistical analyses were carried out using *R*.

### Pattern recognition using artificial neural networks

The Deep Learning tool for MATLAB was used for analysis of the phagocytic activity distribution. The numbers from phagocytic activity distribution data were used to create an input matrix for the neural network. A total of 10 features were used for each of the 19 patients' samples, those being, the average beads per cell, the standard deviation of that average, and the proportion of cells which had internalized 0 through 7 particles making up the remaining 8 columns. Data was then normalized in the range from 0 to 1, where zero represented the lowest value and one represented the highest value. In the target matrix, samples below and above the 2.0 mmol L^−1^ lactate level were denoted as 0 and 1 respectively. Input and target matrices used with the ANNs' can be seen in Table S2.†

MATLABs' pattern recognition and classification function (nprtool) was used to generate a neural network for distinguishing between the high risk and control groups based on their neutrophil phagocytic activity distribution. A two-layer feed-forward network with 10 hidden neurons was used for this purpose as shown in Fig. S4.† This network was then trained using three different training algorithms; scaled conjugate gradient, Bayesian regularization, and Levenberg–Marquardt. For each training algorithm, we trained and tested 100 networks, resulting in a net total of 300 networks. For training and validation, we used data from15 patient samples for each of the 300 networks, whereas, a separate holdout set, consisting of data from 4 patient samples was used for testing the efficacy of these trained networks.

We then obtained the confusion matrices, receiver operator characteristic (ROC) curves along with their respective area under the curve (AUC) values for each of the 300 networks. To get a closer look at the performance of each training algorithm, we also obtained a combined ROC curve and confusion matrix for each training algorithm based on the predictions made by its 100 respective networks.

### Principal component analysis

We also performed principal component analysis on the neutrophil phagocytic activity distribution data set that was used with the artificial neural networks. Data sets with multiple dimensions like ours are common and are difficult to interpret and perceive. Principal component analysis reduces the dimensionality of large data sets while minimizing the loss of information and thereby allows us to interpret the data in a more meaningful way. MATLAB was used for the purpose of implementation of PCA for dimensionality reduction while retaining maximum variance.

## Results

### Phagocytic assay

**Fig. 2:**
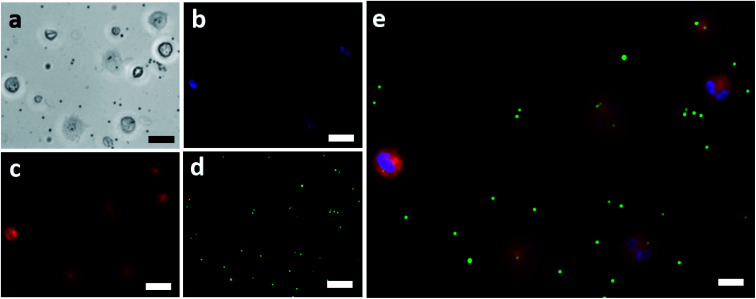
(a) Brightfield image of neutrophils challenged with IgG coated fluorescent beads (scale bar = 20 μm). (b) Nucleus of the neutrophils shown in (a) fluorescing because of the nuclear stain (scale bar = 20 μm). (c) Cellular membranes of the neutrophils shown in (a) fluorescing because of the membrane stain (scale bar = 20 μm). (d) The green IgG coated fluorescent beads used for challenging the isolated neutrophils (scale bar = 20 μm). (e) The final combined imaged showing the neutrophils and the IgG beads that were internalized by each (scale bar = 10 μm).

The isolated neutrophils were exposed to fluorescent IgG coated beads and were imaged using a fluorescent microscope. Fig. 2a shows the bright field image of the isolated neutrophils which were exposed to the IgG beads. These neutrophils were also fluorescently imaged to determine the position of their nucleus and to mark their cellular membrane as shown in Fig. 2b and c, respectively. The IgG beads were also imaged fluorescently as shown in Fig. 2d to determine their position relative to the neutrophils. Lastly, Fig. 2e showcases the final image that was made by merging the three fluorescent images together and confirms the efficacy of the assay used for phagocytic quantification by showing beads internalized by neutrophils. An average of 158 neutrophils was observed from each blood sample. Blood samples in which less than 50 neutrophils were observed were not considered and excluded from the study.

### Phagocytic activity & its distribution

**Fig. 3:**
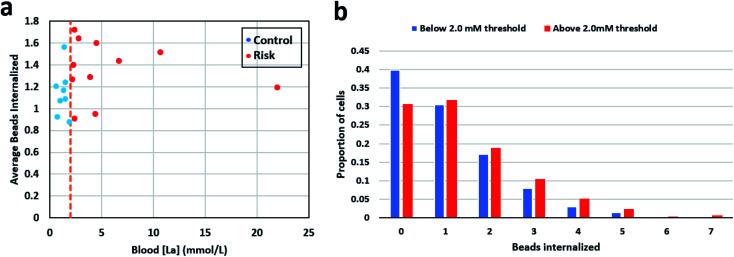
(a) Mean neutrophil phagocytic activity of the patient samples plotted against their blood lactate level. (b) The weighted average neutrophil phagocytic activity distribution for the patient sample in the two groups under consideration.

Mean phagocytic activity for each patient sample was calculated by counting the total number of ingested beads and dividing them by the total number of neutrophils observed for that sample. For samples in the control group (below 2 mmol L^−1^), the mean was 1.14 with a standard deviation of 0.21, and for the high-risk group (above 2 mmol L^−1^), the mean was 1.35 with a standard deviation of 0.26. Fig. 3a shows the mean phagocytic activity for patient samples against their respective blood lactate levels. The visual observation didn't show any distinction among the two groups. Next, we calculated the weighted average phagocytic activity distribution for the two groups under consideration which is shown in Fig. 3b. Single-cell phagocytic activity analysis have shown interesting findings including, (i) a single neutrophil was able to internalize up to 7 beads, (ii) for samples in group below 2 mmol L^−1^, 39.76% cells didn't internalize any beads, while for group above 2 mmol L^−1^, 30.65% cells didn't show any phagocytic activity, (iii) for samples in group below 2 mmol L^−1^, 30.46% cells internalize only 1 bead, while for group above 2 mmol L^−1^, the activity is slightly higher with only 31.73% cells showing a single bead internalization, (iv) however, 7 beads internalization activity was much higher for samples in group above 2 mmol L^−1^ (0.6% cells) compared to group below 2 mmol L^−1^ (0.17% cells). We used multiple statistical methods to test the significance of this difference. In short, patient samples in the control group contained a greater proportion of neutrophils showcasing no phagocytosis. On the other hand, in the high-risk group, neutrophils appeared to be slightly more active, with lower % of neutrophils ingesting no beads, and higher % of neutrophils ingesting greater number of beads. Table S3† lists the detailed summary of all the results compiled for each sample along with its respective lactate level.

### Statistical analysis

Statistical analysis was performed using *R* to evaluate whether there existed a significant difference between the mean neutrophil phagocytic activity of the two groups under consideration. First, Shapiro–Wilk tests were performed to determine the normality of the data in both groups. The Shapiro–Wilk tests yielded the values: *W* = 0.93, *p*-value = 0.48 for the control group, and *W* = 0.95, *p*-value = 0.64 for the high-risk group. The high *p*-values in all the cases indicate that the data does not deviate significantly from normality. Next, we determined if the variances among the data sets are equivalent using Bartlett's test to assess if two-tailed independent *T*-test could be performed. Bartlett's test yielded a *p*-value = 0.53, proving that the variances do not differ significantly. A two-tailed independent *T*-test was subsequently performed and yielded the values (*T*-stat = −1.85, *p*-value = 0.08, *T*-crit = 2.10), highlighting, no significant difference in the mean phagocytic activity of the samples present in the two groups.

### Pattern recognition using artificial neural networks

**Fig. 4:**
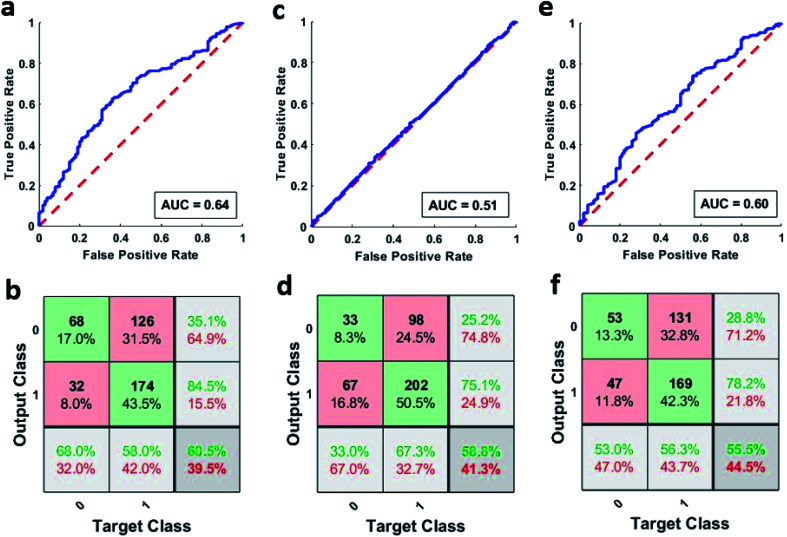
(a) Combined ROC curve of the 100 networks trained using SCG algorithm. (b) Combined confusion matrix of the 100 networks trained using SCG algorithm. (c) Combined ROC curve of the 100 networks trained using BR algorithm. (d) Combined confusion matrix of the 100 networks trained using BR algorithm. (e) Combined ROC curve of the 100 networks trained using LM algorithm. (f) Combined confusion matrix of the 100 networks trained using LM algorithm. (In confusion matrices, 0 represents the control group and 1 represents the high-risk group).

Artificial neural networks were used for classifying the patient samples into control or high-risk groups based on the underlying patterns in their neutrophil activity distribution. MATLAB's pattern recognition tool was used for making a two-layer, feed-forward network with 10 hidden neurons as stated earlier. This network was then trained multiple times using different training algorithms and was tested on a holdout set. Fig. 4a shows the combined ROC curve for the 100 networks that were trained using the scaled conjugate gradient algorithm. A small AUC value of 0.64 means that the networks didn't provide accurate discrimination between the two groups, and AUC is just below the threshold of acceptable discrimination.^21^ However, more extensive studies are needed to further validate this. This can be further seen in Fig. 4b which shows the corresponding combined confusion matrix. The performance of the networks that were trained using Bayesian regularization was also not very different. The combined ROC curves for the 100 networks trained using Bayesian regularization can be seen in Fig. 4c and it also has a meagre AUC value of 0.51. The corresponding confusion matrix shown in Fig. 4d further strengthens this. Furthermore, the networks that were trained using Levenberg–Marquardt also did not fare any better with a combined AUC value of 0.60 as seen from ROC curve shown in Fig. 4e and the corresponding confusion matrix shown in Fig. 4f. The individual AUC values for the 300 trained networks are listed in Tables S4–S6.† The small AUC values mean that the classifiers were not able to detect a significant difference between the phagocytic activity distribution of the samples above and below the sepsis threshold (2 mmol L^−1^).

### Principal component analysis

**Fig. 5:**
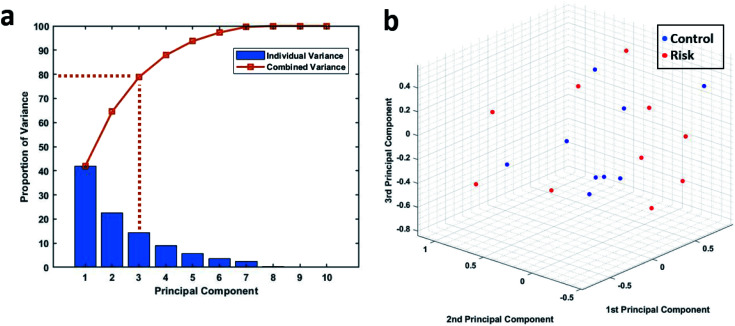
(a) The proportion of variance shared by each principal component along with the combined variance. (b) 3D plot showing the 19 patient samples when plotted on the basis of their first 3 principal components.

Principal component analysis was used to further analyse the phagocytic activity distribution of the isolated neutrophils and MATLAB was used to perform this analysis. Since the data under consideration had a high dimensionality, we first found the proportion of variance that each principal accounted for. Fig. 5a shows the proportion of variance that each principal component accounted for. We considered the first three principal components, since together, they account for almost 80% of the entire variance and allow us to reduce the data's dimension down to 3. The 19 samples were then plotted based on their first three principal components as shown in Fig. 5b. We can see that there is still no clear way to separate the two groups and this further strengthens our earlier results.

## Discussion

The lack of a strong relationship between mean phagocytic activity and blood lactate level is not unexpected, considering the contradicting evidence seen in the phagocytic activity in response to infections and sepsis. While there is general agreement that the overall phagocytic activity rises in patients facing infection, some studies have instead found a decrease. As mentioned before, this may be due to a variety of factors, including the method of measuring and calculating phagocytic activity, and the treatment of data on a cell or a population level, and more importantly the personalized immunological response to the infection. Most of the earlier studies rely on mean of a population while quantifying phagocytic activity which often does little to account for the distribution of phagocytic activity across the cells within each sample. A blood sample with cells exhibiting extreme behavior (*e.g.*, few cells exhibiting high levels of phagocytosis and few with no phagocytosis) could potentially have a similar mean phagocytic activity as that of a blood sample with very high numbers of cells phagocytosing at a moderate rate. Therefore, our single-cell level phagocytosis analysis here extends beyond comparing mean phagocytic index and lactate levels by examining distributions across the different populations.

Distinctions between the normal and at-risk patients are not immediately apparent when looking at the phagocytic activity distributions for individual samples as shown in Table S3.† So, MATLAB's pattern recognition neural network tool was used to distinguish between the two groups. The AUC values indicate weak performance of the neural networks as stated before. Although the combined performance of the 100 networks trained using either of the three training algorithms was lack lustre, individual performances of some of the networks looked very good on a cursory glance, with some networks even reporting a perfect AUC value of 1 as shown in Tables S4–S6†. It is important to note that this does not necessarily point to a difference in the phagocytic activity distribution of neutrophils coming from control and high-risk group. This just means that the hold out test data was very similar to some of the training data for that particular network resulting in an inflated measure of accuracy. To overcome this exact issue, we went ahead with training and testing a multitude of models and reported the combined accuracy. To further validate the findings of our neural networks, we also performed principal component analysis on the same data. Principal component analysis helps us to work when we have too many features compared to the number of samples. Using PCA, we were able to reduce our dimensions down to three while retaining 80% of the total variance but we still were not able to visually see a clear division in the data based on the lactate threshold as shown in Fig. 5b.

Even though neither of the data analysis techniques (*T*-test, artificial neural networks, and principal component analysis) pointed towards a direct relationship between blood lactate level and mean phagocytic activity/phagocytic activity distribution, we cannot rule out a relationship yet with complete certainty. This is because of the certain limitations in our study. In our study, we have just located neutrophils and enumerated the ingested beads without considering the FcR CD16 or CD64+ protein expression levels. The expression of these proteins can have an effect on the phagocytic potential of neutrophils as mentioned before.^10–12^ It is entirely possible that different samples under consideration had different protein expression levels which had an effect on their phagocytic potential. By also accounting for protein expression levels on neutrophils surface, a more robust study can be conducted. Although, our study highlighted interesting phagocytic activity findings at a single cell level, however, it further underscores the importance of the integration of individual patients' medical records to get more personalized insights into individual phagocytic activity in the future. Immune response and phagocytic ability are both dependent on a variety of factors, including age, medical history, underlying conditions, *etc.* Without more knowledge of the age, gender, and pre-existing conditions of the patient population, it is impossible to control for the differences due to natural physiological factors rather than disease states. Additional IRBs are currently underway which will allow for the collection of de-identified patient information, making for a more robust study.

## Conclusions

Neutrophils were isolated from whole blood samples and grouped on the basis of their respective blood lactate levels into control and high-risk group. They were then exposed to IgG coated fluorescent beads for the quantification of their phagocytic activity at a single-cell level. Patient samples in the control group contained a greater proportion of neutrophils showcasing no phagocytosis. On the other hand, in the high-risk group, neutrophils appeared to be slightly more active, with lower % of cells ingesting no beads and higher % of cells ingesting greater number of beads. Based on the collected data and the performed analysis, we were not able to find a strong correlation (AUC < 0.7) between blood lactate levels and mean neutrophil phagocytic activity or the distribution of phagocytic activity in patient neutrophil population. Further improvements to the study would include additional samples to strengthen statistical significance and increase available data for training and validation; increasing the number of neutrophils analyzed per sample; and access to de-identified patient data to control for confounding factors such as age, gender, pre-existing medical conditions.

## Conflicts of interest

U. H. have financial interests in Prenosis, Inc. All other authors declare no competing financial interests.

## Supplementary Material

RA-011-D1RA02759J-s001
