# Supplementary material for: Profiling single-cell level phagocytic activity distribution with blood lactate levels
Source: RSC Adv. 2021 Jun 16;11(35):21315–22. doi: 10.1039/d1ra02759j (PMC9034040; doi:10.1039/d1ra02759j)
Supplement: RA-011-D1RA02759J-s001 [file RA-011-D1RA02759J-s001.pdf]

Electronic Supplementary Material (ESI) for RSC Advances.
This journal is © The Royal Society of Chemistry 2021

Supplementary Information

Profiling Single-Cell Level Phagocytic Activity Distribution with Blood Lactate Levels

Authors: Kurt Wagner[1], Muhammad Ahsan Sami[2], Corey Norton[2], J. McCoy[3], Umer Hassan*[2, 4]

[1] Department of Biomedical Engineering, School of Engineering, Rutgers, The State University of New Jersey, Piscataway, NJ, 08854, USA

[2] Department of Electrical and Computer Engineering, School of Engineering, Rutgers, The State University of New Jersey, Piscataway, NJ, 08854, USA

[3] Department of Emergency Medicine, Robert Wood Johnson Medical Hospital, Rutgers, The State University of New Jersey, New Brunswick, NJ, 08901, USA

[4] Global Health Institute, Rutgers, The State University of New Jersey, New Brunswick, NJ, 08901, USA

*Corresponding Author: umer.hassan@rutgers.edu; Tel (848) 445-2164

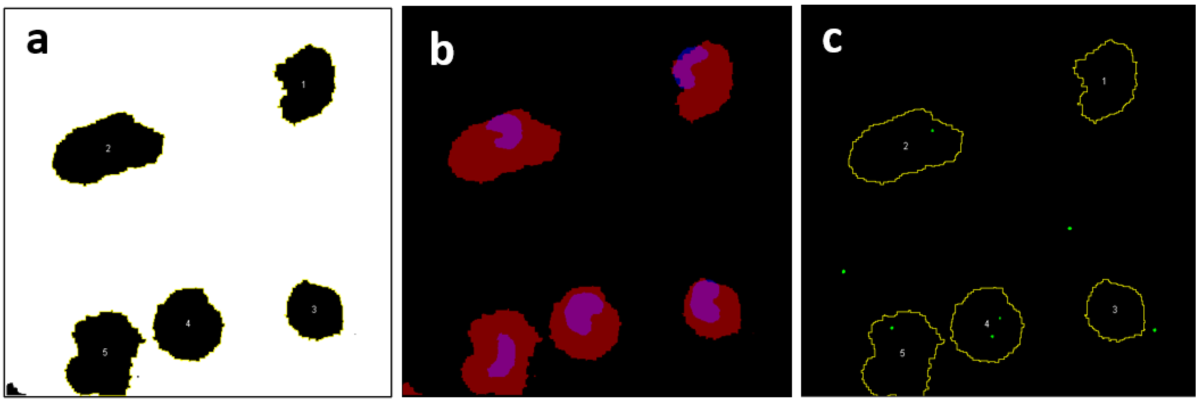

Fig S1. (a) Determines regions of interest (ROI) for future steps. (b) Each ROI is checked for presence of nuclei, to exclude membrane debris. (c) Beads in each region of interest are counted, results are exported to spreadsheet.

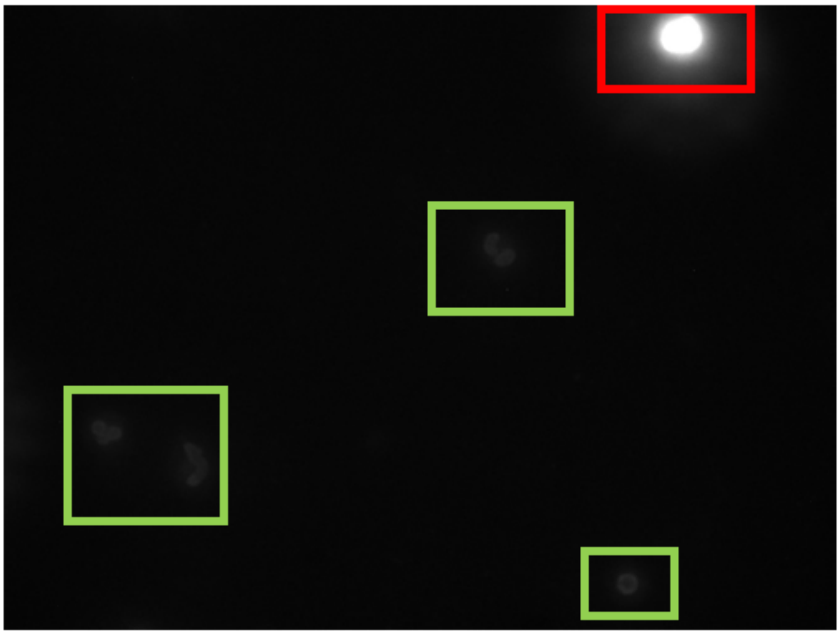

Fig S2. Representative image of excluding dead cells using Hoescht nuclear stain. Nuclei in red box excluded, due to excess fluorescence. Nuclei in green boxes were included in data.

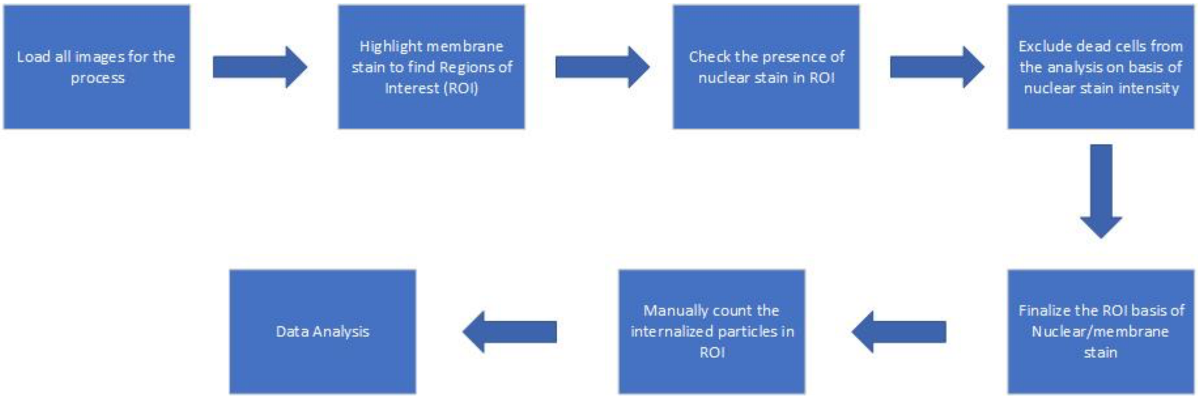

Fig S3. Flowchart listing the steps invloved in processing of the images obtained from the phagocytosis assay for data collection and analysis.

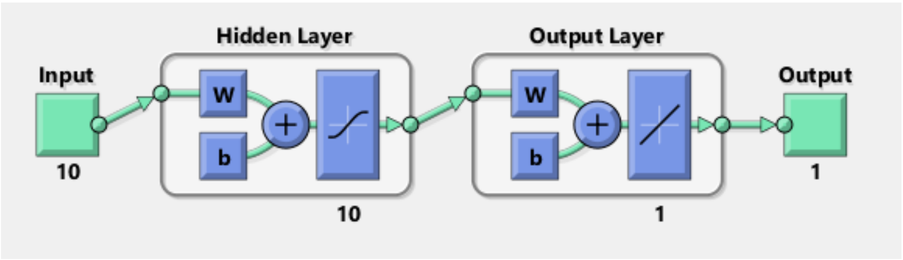

Fig S4. A two layer feed forward network used for differentiating between the phagocytic activity distribution of the neutrophils from the two groups.

Protocol Step	Approximate time requirement
Neutrophil Isolation	Approximately 90 minutes
Neutrophil seeding in 24 well plate	Approximately 15 minutes
Incubation with IgG beads and membrane stain	Approximately 40 minutes
Washing of excess beads and membrane stain	Approximately 10 minutes
Incubation with nuclear stain	Approximately 10 minutes
Placement on ice	Approximately 10 minutes
Washing of excess nuclear stain	Approximately 10 minutes
Fluorescent imaging of the neutrophils	Approximately 30 minutes
Merging captured images using image processing for final image	Approximately 15 minutes per merged image
Data analysis of the final merged image	Approximately 20 minutes per image

Table S1: Approximate time required for each step involved in the protocol used for quantification of neutrophil phagocytic activity.

		Input										Target
Training Data	Sample 1	0.38717	0.561795	0.59679	0.396147	0.41549	0.703447	0	0.374074	0.824074	0	0
	Sample 2	0.053195	0	0.689191	0.669302	0.519734	0.163684	0.07086	0.128662	0	0	0
	Sample 3	0.234294	0.261332	0.632158	0.508699	0.460902	0.440004	0.209906	0.285849	0	0	0
	Sample 4	0.343015	0.447686	0.459527	0.616768	0.770177	0.259615	0.390351	0.177193	0	0	0
	Sample 5	0.814085	0.673964	0.214015	0.609903	0.556113	0.658402	1	0	1	0	0
	Sample 6	0.25108	0.207431	0.565163	0.60309	0.544811	0.441405	0.110697	0.301493	0	0	0
	Sample 7	0.432087	0.57666	0.507983	0.625195	0.448688	0.302067	0.460345	0.557241	0	0.348276	0
	Sample 8	0.460827	0.59076	0.442445	0.889803	0.219538	0.396377	0.393805	0.715044	0.393805	0	1
	Sample 9	0.616471	1	0.559671	0.421688	0.224852	0.437912	0.495668	1	0	1	1
	Sample 10	0.034269	0.189964	0.764805	0.703711	0.252568	0.19514	0.171154	0	0	0.388462	1
	Sample 11	1	0.583481	0	0.609378	0.850268	1	0.628024	0.325806	0.358871	0.407258	1
	Sample 12	0.484942	0.606506	0.448497	0.878604	0.147666	0.297035	0.75282	0.759398	0	0	1
	Sample 13	0.856533	0.772866	0.122608	0.932161	0.540519	0.414394	0.722403	0.787013	0.577922	0.327922	1
	Sample 14	0.754604	0.366946	0.154362	0.511307	1	0.624451	0.728423	0.240476	0	0	1
	Sample 15	0.371917	0.183717	0.362663	1	0.345474	0.617988	0.404545	0	0	0	1
Test	Sample 1	0	0.444159	1	0.372388	0	0.010779	0.526974	0.318947	0	0.265789	0
	Sample 2	0.905046	0.668509	0.266133	0	0.938111	0.873288	0.953571	0.192381	0	0.480952	1
	Sample 3	0.081015	0.588928	0.909807	0.376173	0.270656	0	0.196903	0.357522	0	0.893805	1
	Sample 4	0.662136	0.507851	0.233745	0.777219	0.634291	0.527502	0.533775	0.510101	0.337121	0	1

Table S2: Input and target matrices used with the fro the purpose of training nad testing to evaluate its performance.

Sample Number	Lactate Level	Total cells imaged*	Avg. beads per cell	S. Dev. beads per cell	% cells with 0 bead	% cells with 1 bead	% cells with 2 bead	% cells with 3 bead	% cells with 4 bead	% cells with 5 bead	% cells with 6 bead	% cells with 7 bead
1	0.6	54	1.2	1.35	38.89	27.78	16.67	12.96	0	1.85	1.85	0
2	0.7	157	0.92	1	42.04	32.48	18.47	5.73	0.64	0.64	0	0
3	1	212	1.08	1.16	40.09	29.72	17.45	9.43	1.89	1.42	0	0
4	1.3	114	1.17	1.28	34.21	31.58	22.81	7.02	3.51	0.88	0	0
5	1.4	89	1.56	1.42	25.84	31.46	19.1	12.36	8.99	0	2.25	0
6	1.5	201	1.09	1.13	37.81	31.34	18.91	9.45	1	1.49	0	0
7	1.5	145	1.24	1.36	35.86	31.72	17.24	7.59	4.14	2.76	0	0.69
8	1.9	190	0.88	1.28	52.63	27.37	9.47	3.68	4.74	1.58	0	0.53
9	2.2	113	1.27	1.37	33.63	36.28	13.27	8.85	3.54	3.54	0.88	0
10	2.3	202	1.4	1.63	37.62	28.22	13.37	9.41	4.46	4.95	0	1.98
11	2.4	130	0.91	1.12	44.62	33.08	13.85	6.15	1.54	0	0	0.77
12	2.4	124	1.72	1.36	18.55	31.45	24.19	16.94	5.65	1.61	0.81	0.81
13	2.8	210	1.64	1.42	27.62	20.95	25.71	15.24	8.57	0.95	0	0.95
14	3.9	133	1.29	1.38	33.83	36.09	12.03	7.52	6.77	3.76	0	0
15	4.4	113	0.95	1.37	49.56	27.43	14.16	3.54	1.77	1.77	0	1.77
16	4.5	154	1.6	1.48	22.73	37.01	18.83	9.09	6.49	3.9	1.3	0.65
17	6.7	396	1.43	1.32	26.52	34.34	20.45	10.61	4.8	2.53	0.76	0
18	10.7	169	1.51	1.23	23.81	29.76	26.79	11.9	6.55	1.19	0	0
19	22	110	1.19	1.11	30.91	38.18	15.45	11.82	3.64	0	0	0

Table S3: Table showing the phagocytic activity distributions for individual samples along with their lactate levels.

AUC Values									
0.5	0.5	1	1	1	1	1	1	1	0
0.666667	1	1	1	1	0.666667	0.333333	1	0.666667	1
1	1	1	1	1	1	0.666667	0.5	1	1
1	1	0	1	1	1	1	1	1	1
1	1	1	1	1	0.333333	1	1	1	0.666667
1	1	1	1	1	1	1	0.333333	0.5	1
1	1	1	1	1	0	1	1	1	1
1	1	1	1	1	1	0.666667	1	1	0
1	1	1	1	0.5	0.5	0.5	1	1	1
0.666667	1	0.666667	1	1	1	1	0.5	1	0.333333

Table S4: Table showing the AUC values of 100 networks trained using BR algorithm.

AUC Values									
0.333333	1	1	0.333333	0.333333	1	0.666667	1	1	0
0.666667	0.666667	0.666667	0.666667	0.333333	0.333333	0.666667	0.666667	1	0
1	1	1	1	1	1	0.333333	0.666667	0.333333	0.666667
0.666667	0.666667	0.666667	0.666667	0.666667	0.333333	0	1	1	0.666667
0.666667	0.333333	1	0.333333	0	0	0.333333	1	0.666667	1
0.333333	0.333333	1	0.666667	0.666667	0.333333	1	0.666667	0.333333	1
0.666667	0.666667	0	0.333333	0.666667	0.333333	0	1	0.333333	1
0.333333	0.666667	1	0.666667	0.666667	1	0.333333	0.333333	0.333333	0.333333
1	1	0.666667	0.666667	1	0.333333	0.666667	1	1	1
0	0.666667	0.666667	0.333333	1	0.333333	0.666667	0	0.333333	0.666667

Table S5: Table showing the AUC values of 100 networks trained using LM algorithm.

AUC Values									
1	0.666667	1	0.333333	0	1	0.333333	1	0.666667	0.333333
0.666667	0	0.333333	0.666667	0.333333	0.666667	0.666667	0.666667	1	0.666667
1	0.333333	0.333333	1	0	0.333333	0.666667	0.333333	0.666667	0.333333
0.666667	0.666667	1	0.666667	1	0.666667	1	1	0.666667	1
1	0.666667	0.666667	0.333333	0.666667	0.333333	1	1	0	1
0.333333	0.333333	1	0.666667	1	0.666667	1	0.666667	1	1
0.666667	0.333333	1	0.333333	0.666667	1	0.666667	1	0.333333	0.333333
1	0	0.666667	1	1	0.666667	0.333333	0.666667	0.666667	1
0.333333	1	0.666667	0.666667	1	0.333333	0.666667	0.333333	1	1
0.333333	0.666667	0	1	1	1	0.666667	0.666667	0.333333	0.333333

Table S6: Table showing the AUC values of 100 networks trained using SCG algorithm.
